# Omics Approaches in Understanding Insecticide Resistance in Mosquito Vectors

**DOI:** 10.3390/ijms26051854

**Published:** 2025-02-21

**Authors:** Nikhil Bharadwaj, Rohit Sharma, Muthukumaravel Subramanian, Gnanasekar Ragini, Shriram Ananganallur Nagarajan, Manju Rahi

**Affiliations:** Division of Vector Biology and Control, ICMR-Vector Control Research Centre, Medical Complex, Indira Nagar, Puducherry 605006, India; kumaravelmuthuvel@gmail.com (M.S.); ragisarosekar99@gmail.com (G.R.); anshriram@gmail.com (S.A.N.); drmanjurahi@gmail.com (M.R.)

**Keywords:** insecticide resistance, omics, vector-borne diseases, mosquitoes, systems biology

## Abstract

In recent years, the emergence of insecticide resistance has been a major challenge to global public health. Understanding the molecular mechanisms of this phenomenon in mosquito vectors is paramount for the formulation of effective vector control strategies. This review explores the current knowledge of insecticide resistance mechanisms through omics approaches. Genomic, transcriptomic, proteomic, and metabolomics approaches have proven crucial to understand these resilient vectors. Genomic studies have identified multiple genes associated with insecticide resistance, while transcriptomics has revealed dynamic gene expression patterns in response to insecticide exposure and other environmental stimuli. Proteomics and metabolomics offer insights into protein expression and metabolic pathways involved in detoxification and resistance. Integrating omics data holds immense potential to expand our knowledge on the molecular basis of insecticide resistance in mosquitoes via information obtained from different omics platforms to understand regulatory mechanisms and differential expression of genes and protein, and to identify the transcription factors and novel molecules involved in the detoxification of insecticides. Eventually, these data will help construct predictive models, identify novel strategies, and develop targeted interventions to control vector-borne diseases.

## 1. Introduction

Omics is a vast area referring to the interdisciplinary application of various high-throughput screening technologies to study DNA, RNA, proteins, and metabolites. They are represented by genomics, transcriptomics, proteomics, and metabolomics, respectively. The area of omics has been widely used in identifying several genes and pathways that are crucial for biological processes under various conditions; therefore, omics has high applicability in the field of biotechnology and biomedicine [1,2,3]. The use of omics in vector-borne diseases has been demonstrated for studying conditions like emerging insecticide resistance in vector mosquitoes, host–pathogen interactions, and enhanced susceptibility to various infections [4,5]. There are several genes differentially expressed during vector/pathogen/host interactions and may play important roles in vector competence [6]. Omics approaches can be useful in the characterization of such important biological pathways and processes. Data obtained can eventually help health professionals with the required knowledge to understand the transmission dynamics of vector–pathogen interaction. The emergence of insecticide resistance in mosquito populations is a global problem leading to the resurgence of mosquito-borne diseases [7]. The mosquito vectors develop favorable adaptations and undergo extensive selections under repeated insecticide exposure. This results in the emergence of resistance factors and novel mechanisms in vectors. These resistant mechanisms have been extensively studied and explored [8,9]. In the future, omics can be helpful in decision-making for comprehensive health and wellness assessment in vector-borne diseases and insecticide resistance surveillance programs.

Globally, of the 91 malaria-endemic countries, 62 countries have reported the presence of resistance to at least one class of insecticide, and 50 countries have shown evidence of resistance to two or more classes of insecticides [10]. The failure to mitigate and manage insecticide resistance can lead to increased disease burden and, thus, may potentially reverse the gains and efforts of control programs. Insecticide resistance may alter the vector competence of the vector. This may be due to the expression of genes that are involved in blood feeding and acquiring immunity [11,12,13]. These biological changes can alter the longevity of the vectors [14,15]. Research has shown that insecticide resistance in mosquito vectors is acquired by the presence of two primary mechanisms, increased detoxification of the insecticides by overexpression of inherent detoxification enzymes and through favorable mutations, which result in reduced sensitivity of target sites to applied insecticides (target-site resistance) [16]. Cuticular thickness (penetration resistance) and behavioral avoidance have also been implied for the development of resistance towards insecticides in mosquito vectors [17,18,19]. An inclusive understanding of resistance mechanisms and regulations in vectors needs to be studied carefully. The recent advances in omics can enhance our understanding of associated molecular mechanisms of insecticide resistance in mosquito vectors and facilitate the development of newer insecticides in the future. However, the characterization of phenotypic resistance in vector mosquitoes is essential in the determination of inherent resistance mechanisms related to target-site and metabolic resistance. This review aims to highlight the application of omics towards understanding insecticide resistance in vector mosquitoes.

### 1.1. Important Mosquito Vectors and Vector-Borne Diseases

**Figure 1:**
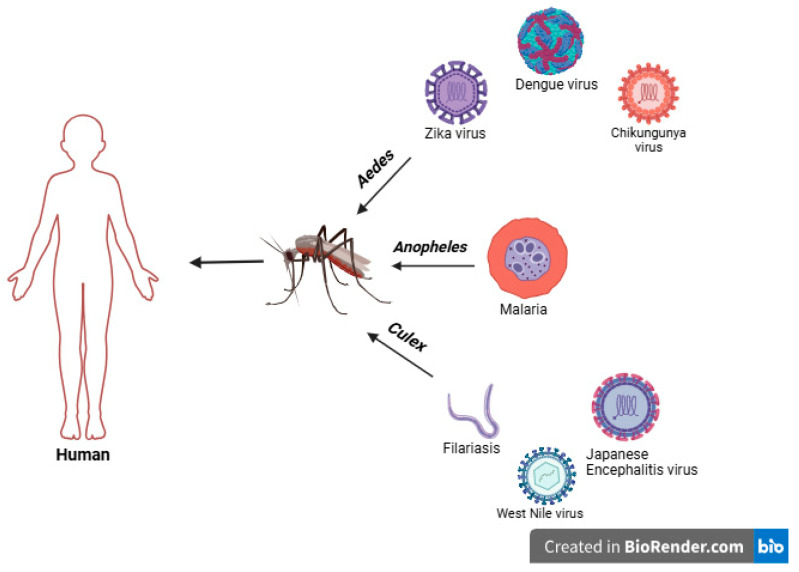
Major mosquito-borne diseases.

Vectors are bloodsucking arthropods (such as mosquitoes and ticks) capable of acquiring, harboring, and transmitting disease-causing pathogens (such as viruses and parasites) between hosts, including animals and humans. Vector mosquitoes ingest viruses and parasites during the blood meal from the infected host. The pathogen then starts replicating (in the case of arboviruses) or developing into infective stages (in the case of parasites) within the vectors. It is transmitted to a new host with each subsequent bite/blood meal for the rest of their lives [20]. Vector-borne diseases are widespread and are caused by a range of vectors like the *Aedes*, *Culex*, and *Anopheles* species of mosquitoes (Figure 1). According to one of the estimations carried out by the World Health Organization (WHO), more than 700,000 deaths take place every year from vector-borne diseases like malaria, dengue, chikungunya, yellow fever, and zika. The deprived populations are disproportionately affected by these diseases, which are most prevalent in tropical and subtropical regions [21,22]. The repercussions are severe, claiming many lives, and have overwhelmed public health systems in many countries. Diseases like chikungunya, leishmaniasis, and lymphatic filariasis cause long-lasting suffering, lasting morbidity, disability, and societal stigmatization [22]. An intricate set of demographic, societal, and environmental factors play an important role in the spread of vector-borne diseases [23]. Global travel, trade, and unplanned urbanization are other important contributing factors for the spread of vector-borne diseases [22,24].

### 1.2. Rising Threat: The Impact of Insecticides on the Surge of Resistance in Mosquito Populations

Global warming and associated climate changes are leading to rapid evolutionary adaptations in vectors causing vector-borne diseases worldwide (Table 1) [25,26]. The heritable evolutionary changes and constant exposure to the insecticides make the mosquitoes insecticide resistant, posing a threat to the existing mosquito control programs worldwide and, thus, increasing the risk of the spread of vector-borne diseases [27]. Among the many vector control techniques followed, the application of insecticide has been the cornerstone of vector control programs in India and across the globe [28]. To control vector mosquitoes, the following insecticides are currently used in India. For controlling adult mosquitoes, dichloro-diphenyl-trichloroethane (DDT), malathion, and pyrethroids (deltamethrin, lambda-cyhalothrin, bifenthrin, alpha-cypermethrin, and cyfluthrin) are used. For larval control, temephos, bacterial toxins (*Bacillus thuringiensis* and *Bacillus sphericus*), and insect growth regulator (IGR) compounds—pyriproxyfen and diflubenzuron—are used. Space spraying of malathion, deltamethrin, cyphenothrin EC, and pyrethrum extract formulations have been routinely applied to control adult mosquito populations [29].

### 1.3. Current Landscape of Insecticidal Resistance Status in Mosquito Vectors

The widespread emergence of insecticide resistance has become a major hurdle for malaria control programs in sub-Saharan Africa. In coastal Kenya, *An. arabiensis* is the predominant vector, which accounts for approximately 95.2% of cases, followed by *An. gambiae* at around 4.8% [30]. *An. arabiensis* has developed resistance to DDT and pyrethroids, which are commonly used in indoor residual spraying (IRS) and long-lasting insecticidal nets (LLINs) in African vector control programs [31]. *An. gambiae* has developed resistance to various synthetic pyrethroids, such as deltamethrin and permethrin, as well as organophosphate and carbamates [32,33]. Furthermore, *An. stephensi* has increasingly adapted to urban environments [34]. In West, Central, and East Africa, *An. funestus* has shown reduced susceptibility to pyrethroids [35].

In India, malaria transmission is predominantly driven by *An. culicifacies*, accounting for approximately 67% of cases in rural and peri-urban areas. *An. fluviatilis* is responsible for about 15% of malaria cases in forested regions, while *An. stephensi* contributes to roughly 12% of cases in urban areas [36]. Resistance to DDT (4%) and malathion has been documented in *An. culicifacies* populations across several districts in Madhya Pradesh, as well as in tribal regions of Andhra Pradesh, Odisha, Jharkhand, and West Bengal [37,38].

*Ae. aegypti*, a primary vector for several arboviral diseases such as dengue, chikungunya, zika, and yellow fever has shown developing resistance to all major neurotoxic insecticides across America and Asia; although, data from Australasia and Africa remain insufficient. Resistance to carbamates, organochlorines, organophosphates, and pyrethroids is emerging, with particularly high resistance to deltamethrin in parts of South America, including Brazil and French Guyana. In southeast Asia, resistance patterns are more variable and uneven [39]. In India, *Aedes* species have shown high levels of resistance to DDT and widespread resistance to carbamates. Increased tolerance to pyrethroids and organophosphorus compounds such as permethrin, deltamethrin, lambda-cyhalothrin, malathion, and temephos has also been observed [40].

The insecticide resistance status of the filarial vector *Culex* species has been extensively studied worldwide. In Africa, *Cx. quinquefasciatus* has exhibited resistance to permethrin (with mortality rates ranging from 4 to 24%), deltamethrin (24 to 48%), DDT (4 to 12%), and bendiocarb (60–76%) in Benin [41]. The *Cx. pipiens* complex in Europe has shown widespread resistance to various insecticides, with populations in Belgium exhibiting resistance to deltamethrin, permethrin, DDT, and possible resistance to bendiocarb [42]. In Brazil, *Cx. quinquefasciatus* has been reported to resist organophosphates, carbamates, DDT, and pyrethroids [43]. Studies in India have revealed multiple resistances in *Cx. quinquefasciatus* to various insecticides, including deltamethrin, lambda-cyhalothrin, permethrin, DDT, propoxur, and malathion [44].

### 1.4. Exploring the Mechanisms: How Mosquito Vectors Develop Insecticide Resistance

**Figure 2:**
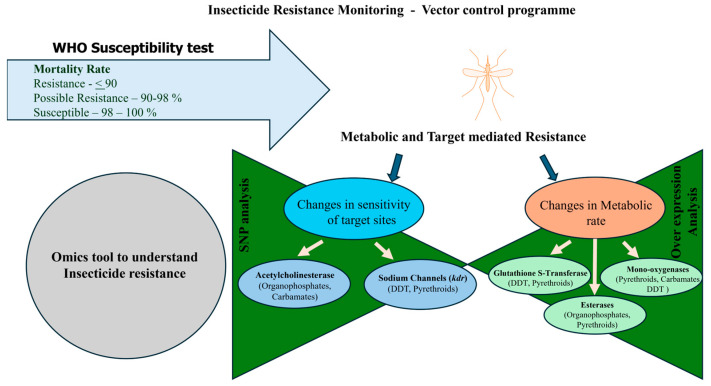
Insecticide resistance monitoring—vector control program.

Mosquitoes are highly evolved arthropods with the ability to develop resistance to insecticides. These biological adaptations enable them to survive in environments treated with insecticides. Several mechanisms contribute to the development of insecticide resistance (Figure 2). These include (a) behavioral resistance, (b) target-site mutations, (c) metabolic resistance and sequestration, and (d) cuticular resistance. Behavioral resistance involves changes in mosquito behavior and perception, leading them to avoid areas with insecticides [17,45]. Target-site mutations occur due to point mutations in genes encoding proteins targeted by insecticides and prevent them from binding or interacting at their intended site of action [46,47,48]. Cuticles, composed of chitin polysaccharides and proteins, thicken in response to insecticides, hindering penetration of applied insecticides [18,49]. Metabolic resistance arises from gut microbiome detoxification and inherent detoxification systems present in the vector [50]. Monitoring and understanding these mechanisms are helpful in integrated surveillance and mosquito control programs. Eventually, this in turn helps to design better insecticide formulations for controlling these diseases transmitting vectors.

## 2. Omics and Systems Biology as a Tool to Understand Insecticidal Resistance

**Figure 3:**
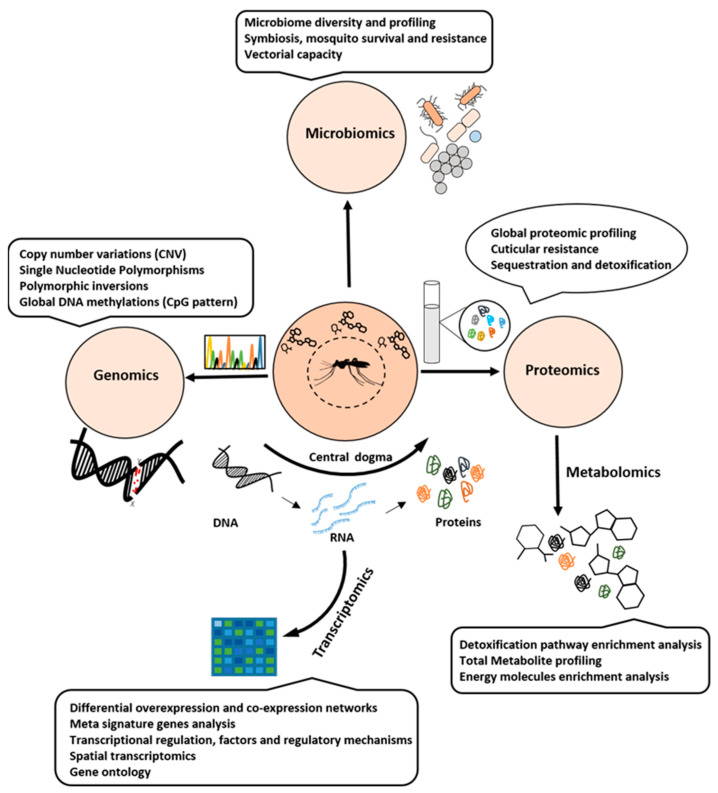
Overview of the application of omics approaches in insecticide resistance.

Omics play an important role in facilitating the understanding of the molecular mechanisms underlying various biological processes [51]. It provides insights into genetics, gene expression, differential expression of proteins, and metabolomic changes associated with the complex biological process like insecticide resistance (Figure 3). Genomics can be studied through approaches like copy number variations (CNVs), single-nucleotide polymorphisms (SNPs), polymorphic inversions, and global DNA methylation patterns. Transcriptomics focusses on differential expression and co-expression networks, meta-signature analysis, transcription factors, regulation, and other related mechanisms. Proteomics studies enable global proteomic profiling and provide insights into cuticular resistance, sequestration, detoxification, and other related mechanisms. Similarly, metabolomics has become a valuable tool for detoxification pathway enrichment analysis, total metabolite profiling, and energy molecule enrichment analysis.

Comparative omics is a subfield within the broader omics domain. It involves layered comparisons of genes, regulatory mechanisms, introns, and exons. These data provide insights into evolutionary relationships and the conservation of different functions between species [52]. The layered omics approach helps to compare and understand relationships between genome structure, protein function, and associated complex pathways in the biological system [53,54,55]. With multilayered omics data, complex relationships can be understood at various biological levels, illuminating how genetics influence the relationships between proteins, metabolites, gene expression, and epigenetics and thus help in understanding cellular processes and molecular pathways. This principle can be used to understand the mechanisms underlying the development of insecticide resistance in mosquitoes [4]. Comparative proteomics can contribute to functional genomics and assist in characterizing the function of unknown genes/proteins involved in novel biological mechanisms [56]. It can offer knowledge of protein expression patterns, post-translation modifications, and interactions across various species of mosquitoes [53,54]. This tool can be used to annotate resistance-specific biomarkers for early surveillance and monitoring of the development of insecticide resistance in the populations of mosquito vectors. Comparative metabolomics can also help with the understanding of the evolutionary trends and detoxification pathways between different species. Overall, each branch of omics provides unique clues and helps in understanding the bio-molecular architecture and system.

Systems biology plays a pivotal role by integrating diverse omics data to provide a holistic view for understanding novel and complex biological mechanisms [51]. It employs computational and mathematical approaches to integrate complex omics data. This integration allows researchers to map cellular processes and their regulation [57]. Integrating data from genomics and transcriptomics can be used to establish a connection between genetic differences and changes in gene expression when the development of resistance mechanisms is due to metabolic resistance [4]. Adding data from proteomics and metabolomics can make it more clear as to how these changes impact these detoxification enzyme functions, metabolic pathways, and the metabolites formed in the process. System biology can also help in building and analyzing biological networks based on the available omics data. These networks offer insights into the functional links and pathways that govern cellular behavior and help in understanding the relationships among genes, proteins, metabolites, and other components of biological pathways. Examples of these networks are metabolic, gene regulatory, and protein–protein interaction networks [58].

Multi-omics data can enhance the precision and comprehensiveness of these networks, enabling the identification of key regulatory pathways involved in differential gene expression. By integrating multi-omics data, researchers can unravel possible mechanisms of resistance. Merging genomic data with epigenomic and transcriptomic information can show how genetic mutations and epigenetic changes impact gene expression and lead to resistance [59]. Likewise, combining proteomic and metabolomics data can provide information on how these alterations influence cellular metabolism and overall phenotypes [60]. Data integration can also help in the identification of biomarkers that are more specific and sensitive than those derived from a single omics approach [61]. Overall, by mapping the effects of genetic mutations, expression changes, metabolic pathway alterations, critical nodes, and pathways, targeted strategies may be designed to control and monitor the development of insecticide resistance.

Predictive modelling/mapping can offer a great advantage for public health scientists in surveillance programs. These models can be built on the stimulation of the effects of genetic, environmental, and other factors playing a critical role in the evolution of resistance. They can help predict the development of insecticide resistance and guide early control measures. Detailed models and networks can assist in developing more precise surveillance protocols to examine the development and evolution of insecticide resistance in respective demographic setups [62]. Systems biology can assist in experimental design and hypothesis testing by offering a framework for the integration and interpretation of omics data [63]. Thus, systems biology integrated with omics is an advantage and helps in the study of the development of resistance and its control.

### 2.1. Variation in Resistance Mechanisms Across Mosquito Species

Resistance mechanisms can vary across vector species due to significant variations in behavior, ecology, and genetic responses to applied insecticides. *Culex* often breeds in polluted water and is more exposed to industrial and agricultural chemicals [64]. This variation in breeding behavior may facilitate the *Culex* species in developing resistance via a metabolic-mediated detoxification system rather than target-site resistance. *Anopheles* species which often breed in clean water are less exposed to xenobiotics and industrial pollutants and are more exposed to insecticides used in the human dwelling for malaria control programs, facilitating in their development of *kdr*-based mechanisms. However, a study by Huzortey and team found *An. coluzzii* breeding expansion to polluted water and its co-existence with *Culex* species [65]. *Culex* species have a high reliance on esterase (esterase A2 and B2) and cytochrome P450s (CYPs) overexpression for the detoxification of organophosphates, carbamates, and pyrethroid [66]. Metabolic resistance is dominated by overexpression of P450 mono-oxygenase in all the important vector species for the detoxification of pyrethroids. *Ace-1* mutations (G119S), which confer resistance to organophosphates and carbamates, are predominant in *Culex*, less commonly reported in *Anopheles*, and they are very rare in *Aedes*. While enhanced overexpression of glutathione S-transferase (GSTs) for the detoxification of organophosphate and DDT is observed in *Culex*, *Anopheles*, and *Aedes* species. There exists a common occurrence of *kdr* mutations in *Aedes* and *Anopheles*; however, it is less common in *Culex* [67]. Feeding time, resting behavior, and host preferences differ among species. These differences may also contribute to the variations in the evolution of insecticide resistance. Understanding these critical differences is a crucial part of the formulation of a vector control strategy, and omics can help in deciphering the same. Omics can be helpful in understanding the modalities of insecticide resistance mechanisms across species. The differences are reflected in transcriptomics and proteomics data and, thus, may help in understanding the prevalent molecular mechanisms present in respective vector species.

### 2.2. Genomics

The genetic foundation of insecticide resistance has been studied for over 60 years [68], but current developments in DNA sequencing technology can revolutionize our understanding of complex resistance mechanisms. Genomics can provide valuable insights to define and distinguish the resistance and susceptibility status of insect populations. It can be studied under various important domains like SNPs, CNVs, polymorphic inversions, global DNA methylation, and Cystosine-phosphate-Guanine (CpG) patterns. The use of genomics may help to explain the molecular basis of insecticide resistance resulting from selection pressure due to several biotic and abiotic factors acting on the vectors. Mosquitoes continuously evolve in terms of defense mechanisms against insecticides. With advanced techniques like whole-genome sequencing and oligo-array gene chips, it is now possible to explore genome-wide changes in genes that may be involved in resistance mechanisms against specific insecticides. The use of genomic tools for tracking the distribution and surveillance of insecticide-resistant vectors can be very useful in addition to studying phenotypic bioassays of resistance.

Understanding genetic changes in genes, like *vgsc* (the voltage-gated sodium channel: pyrethroid and DDT target), *Rdl* (resistance to dieldrin,gamma-aminobutyric acid: dieldrin target), *GSTs* (glutathione S-transferase: detoxification enzyme/metabolic resistance), and *Ace-1* (acetylcholinesterase: carbamate target), of resistant vectors can be useful in the surveillance of genomic polymorphisms linked to the specific insecticide resistance [46,69,70]. An ample amount of data has been generated using whole-genome sequencing. These sequence data are continuously contributing to the understanding of the molecular foundation of the emergence of insecticide resistance. For example, a study was conducted with the *vgsc* gene, a molecular target of pyrethroids and DDT insecticides [48,71]; in this work, a consortium of many *An. gambiae* specimens from a wide geographical range was sequenced to study the natural mosquito populations. This work discovered forty-seven new protein-altering modifications within the *vgsc* gene, out of which seventeen were at appreciable frequency in one or more populations, and they appeared to be under selection for insecticide resistance [72]. Initially, the *vgsc* gene was studied using targeted capillary sequencing of exons and introns. These studies have characterized molecular characteristics of *kdr* gene [73], identification of resistant variant L1014S through allele-specific kdr diagnostics tool [47], and secondary variant in *An. gambiae*, which substantially enhanced the resistance phenotype of L1014F [74,75].

Yi and co-workers reported resistance to multiple insecticides in *Ae. albopictus* from Hainan province, China. Pre-exposure of mosquitoes to synergist piperonyl butoxide (PBO) increased mosquito mortality in the range of 2.4–43.3%. However, they identified significant *kdr* mutations, F1534S in both susceptible and resistant populations, indicating that F1534S mutations cannot be solely used to predict resistance status [76]. Hernandez and co-workers studied the impact of V410L mutations on the probability of survival of *Ae. aegypti* (L.) when exposed to the insecticide permanone (permethrin and PBO mixture) in Harris County, Texas, USA. They observed that the mosquitoes from these areas were triple-resistant for the V410L, V1016I, and F1534C genotypes (tri-locus homozygous). The V410L mutation added an increased risk factor to the control of deltamethrin-resistant mosquitoes [77].

Park and co-workers conducted a study to investigate the relationship between insecticide resistance and target-site mutation (L1014 *kdr* and G119 *Ace* alleles in five vector species, *Aedes vexans*, *Ae. albopictus*, *Anopheles* spp., *Culex pipiens* complex, and *Culex tritaeniorhynchus*) from six collection sites in South Korea. They could observe the presence of L1014 mutations in all species studied except *Ae. vexans* and *Ae. albopictus*. They also found a correlation between the resistant phenotype and the presence of L1014 and G119 *Ace* mutations only in the *Anopheles* spp. population [78]. 

Dykes and co-workers studied the significance of *kdr* mutations L1014F and L1014S in an Indian population of *An. culicifacies*. They observed that both *kdr* mutant alleles were mostly found in heterozygous conditions without deviating from the Hardy–Weinberg equilibrium. They could conclude that these mutant alleles showed protection against deltamethrin, whereas only the L1014S mutation showed protection against DDT when tested separately [79]. 

A pyrethroid-resistant *Ae. aegypti* population with V1016G and F1534C mutations was observed and tackled a with PBO synergist in Laos [80]. Kasai and co-workers stressed the suppression of permethrin penetration through the cuticle of resistant mosquitoes having V1016G and F1534C mutations [81]. In Grand Cayman, DDT- and pyrethroid-resistant *Ae. aegypti* populations showed overexpression of Glutathione S transferase, cytochrome P450, and esterases levels associated with *kdr* mutations in the voltage-gated sodium channel (V1016I in domain II, segment 6 and F1534C in domain III, segment 6) [82]. The presence of a *kdr* mutation was identified in the pyrethroid-resistant *Ae. aegypti* population during a pesticide resistance screening program in California, USA, and the specific mutation was identified in the sodium channel gene (V1016I and F1534C) in 2017 [83]. Brengues and co-workers discovered a *kdr* mutation with a substitution in the S6 hydrophobic section of domain II of *vgsc* that results in target-site resistance [84]. Many other studies related to target-site resistance in *vgsc* and other genes were studied, and important findings on different vectors have been collectively tabulated in Table 2.

Before the introduction of whole-genome sequencing (WGS) studies, only a few of the genes/mutations responsible for the development of insecticide resistance were known. With the advent of WGS, population structure and evolutionary dynamics can be studied, thus offering a holistic view of how resistance spreads and evolves [85]. WGS has helped in associating the *Ace-1* gene with the development of insecticide resistance. The *Ace-1* gene was found to be the target of carbamate and organophosphate insecticides. A G119S mutation was found to be associated with the development of resistance to these insecticides in *An. gambiae* populations [86,87]. Rahayu and co-workers detected an *Ace-1* mutation in F290V and F455W along with a point mutation at codon 506 without changing the amino acid it is coding for among the temephos-resistant *Ae. aegypti* population in West Sumatra, Indonesia [88].

The gamma-aminobutyric acid locus subunit encodes mutations leading to resistance to the dieldrin class of insecticides [89]. Due to its slow environmental degradation, the usage of dieldrin has been banned. Despite the ban on its usage in control programs, the mutations have persisted for generations in malarial vector populations. A296S V327I SNPs in the *Rdl* locus were observed in malarial vector *An. funestus* in the African subcontinent [90]. Yang and co-workers detected SNPs A296S, V327I, and T345S in a dieldrin-resistant population of *An. sinensis* in Guangxi, China. However, A296S was the most abundant mutation observed [91].

The effect of SNPs on metabolic resistance and the development of insecticide resistance was studied through mutations observed in the *GSTe2* gene (*glutathione S-transferase epsilon 2*). Through genome-wide transcription analysis, Riveron and co-workers identified the *GSTe2* gene to be the most overexpressed gene in DDT- and permethrin-resistant mosquitoes from Benin. SNP analysis established a point mutation L119F strongly associated with the detoxification of DDT and permethrin [92]. The *GSTe2* I114T mutation was found in all *An. gambiae sensu lato* species (*An. arabiensis*, *An. gambiae s.s.*, *An. coluzzii*, and *An. gambiae-coluzzii* hybrids) conferring resistance to DDT [93].

The interesting phenomena of CNVs are a form of genetic variations in which some of the gene sequences in the genome are repeated or deleted. The number of these variations that are occurring varies between individuals of the same species. They affect the expression, structure, and functional levels of coding sequences and thus play a crucial role in evolution and adaptation [94,95,96]. Duplication events in mosquitoes are observed in important insecticide target genes like *vgsc* [97] and *Ace-1* [98]. CNVs were found to provide for the development of insecticide resistance through increased gene expression of resistant genes or permanent heterozygosis (carrying two different alleles) which allows it to escape from the fitness costs of carrying resistance mutations in the absence of applied insecticides. The *Ace-1* gene follows this mechanism to confer insecticide resistance in some mosquitoes [98]. The *Ace-1* gene duplications were observed in *Cx. pipiens* species, involving the SNPs G119S or F290V. Permanent heterozygotes were created through a tandem event involving the linkage of a resistant and wild-type copy with a probability of no recombination events occurring between copies. This duplication event led to the exhibition of resistance to carbamate and an organo-phosphate group of insecticides and a reduction in fitness costs [99]. Recently, Lucas and co-workers have discovered a strong selective sweep tied to CNVs involved in the development of non-pyrethroid insecticide resistance (primiphos-methyl) in the *COEAE2G-COEAE6G* cluster of carboxylesterase genes. The copy number at this locus was found to be significantly associated with primiphos-methyl resistance. Similarly for deltamethrin resistance, they found a novel CNV allele in the *CYP6AA/CYP6P* cluster [100].Through the whole-genome sequencing of several samples from the *An. gambiae* 1000 genome project, multiple CNVs were found across several clusters. Considerable enrichment of the clusters was observed in gene families linked to metabolic resistance [101].

Next-generation sequencing (NGS) is a revolutionary technique that is being used in genomics. It enables rapid and high-throughput sequencing of DNA and RNA. Kothera and co-workers used targeted NGS and recognized genetic variations in southern US populations of *Cx. quinquefasciatus* that are linked to insecticide resistance. They could identify several genetic changes that are linked to insecticide resistance by analyzing SNPs and CNVs. The studies revealed some 228 unique SNPs with significant *p*-values out of which 144 SNPs were associated with malathion resistance and 84 SNPs were associated with permethrin resistance. These SNPs (around 50%) were non-synonymous since they resulted in changes to the amino acid sequence of the encoded proteins. These non-synonymous SNPs were associated with genes such as cytochrome P450, esterase, and glutathione-S-transferase. Around 16 genes (genes belonging to the family of *GST*, *P450*, *chaperonin*, and *vgsc*) were observed to have increased copy numbers and were associated with resistance to malathion and permethrin. Notably, the L1014F *kdr* mutation and additional mutations in the acetylcholine esterase gene were found, which is known for its involvement in conferring resistance to organophosphate insecticides [102].

Transcriptomics (RNAseq) coupled with WGS techniques revealed several changes at the transcriptome and the genome level of major malaria vector *An. gambiae*, which involved 2Rb inversion. The 2Rb inversion is one of the polymorphic inversions on chromosome 2, the other being 2La [103]. This genetic phenomenon may facilitates the ability of mosquitoes to thrive in diverse conditions and contributes to population structure.

Target multiplex amplicon assays coupled with next-generation sequencing technologies were used for sequencing several insecticide-resistance genes from a mosquito population. The methods provided an innovative and high-throughput approach for screening target regions of interest in large datasets [46]. A surveillance tool called the amplicon-sequencing (Amp-seq) method was developed to characterize pathogens at the interface of their interaction with vector mosquitoes (*P. falciparum* and *An. gambiae*) [104]. To characterize the loci linked to insecticide resistance in *An. stephensi*, *vgsc*, *Rdl*, *GSTE2*, and *Ace-1*, Palmer and colleagues created and validated the Amp-seq bioassay in conjunction with dual index barcodes and Illumina sequencing. They could determine non-synonymous SNPs that had never been identified in any mosquitoes, in addition to the known SNPs linked to insecticide resistance. This demonstrated the technology’s potential for researching both known and novel mutations that could contribute to the emergence of insecticide resistance. This study validated the use of Amp-seq in monitoring insecticide resistance and exploring genetic diversity at the same time [69]. However, the SNPs need to be further characterized to ascertain the nature of their phenotype (homozygous/heterozygous) upon exposure to insecticides, which results in strong or weak responses.

**Table 2:** Major target-site mutations observed in different mosquito vectors.

Scientific Name	Common Name	Mutation	Transmembrane Domain/Gene	Ref.
*Aedes aegypti*	Yellow fever mosquito	V1016I	II	[82,83]
		V1016G	II	[81]
		F1534C	III	[82,83]
		L982W	II	[84]
		V410L	I	[105]
		I1011M	II	[84]
		I1011V	II	[106]
		S989P	II	[107]
		G923V	II	[84]
		T1520I	III	[108]
		D1794Y	IV	[109]
		F1269C	III	[110]
*Aedes albopictus*	Asian tiger mosquito	I1532T	II	[111]
		F1534L	III	[111]
		F1534S	III	[111]
		F1534C	III	[112]
		V1016G	II	[113,114]
*Anopheles gambiae*	African malaria mosquito	G119S	*Ace-1*	[115]
		L1014S	II	[34]
		A296G	*Rdl*	[116]
		A296S	*Rdl*	[116]
		N1575Y	III-IV	[74]
		V402L	I	[117,118]
		L995F	II	[119]
		N1575Y	III/IV	[74]
		I1527T	III	[117]
		I114T	*GSTe2*	[93]
*Anopheles culicifacies*	Asian malaria mosquito	V1010L	II	[120]
		L1014F	II	[120]
		L1014S	II	[120]
*Culex quinquefasciatus*	Southern house mosquito	L1014F	II	[121]

### 2.3. Transcriptomics

Transcriptomics is the detailed analysis of an organism’s transcriptomes comprising the complete set of RNA transcripts produced during specific biological processes at a given time. It gives an inclusive view of the types of RNA transcripts produced during certain biological processes of importance like the development of insecticide resistance in response to applied insecticides. Transcriptomics can be extensively applied to understand insecticide resistance by interpreting the differential expression of certain genes and ascertaining transcription regulatory factors responsible for the upregulation/downregulation of certain genes involved in the development/modulation of insecticide resistance mechanisms.

Transcriptomics studies have identified several genes belonging to the class of cytochrome P450 oxidases involved in the development of insecticide resistance (metabolic resistance). Several genes of this family have been identified to be differentially overexpressed when compared to those of susceptible strains [16,81,111,122,123]. Martins and co-workers identified two new candidate genes *CPIJ020018* and *CYP6N23* belonging to the cytochrome P450 gene families in transcriptomics analysis. They were significantly upregulated in bendiocarb-resistant *Cx. quinquefasciatus* mosquitoes [124]. The transcriptional overexpression of *CYP6AA7* was seen in pyrethroid-resistant strains of *Cx. quinquefasciatus* across all the life stages [125]. Kwiatkowska and co-workers revealed that *An. gambiae s.s* from Vallée du Kou, Burkina Faso, showed metabolic resistance mechanisms with a prevalence of high frequencies of L1014F and *Rdl* mutations. cDNA microarray data revealed that *CYP6P3* and *CYP6Z2* genes associated with pyrethroid resistance were transcriptionally overexpressed in these adult *An. gambiae* female mosquitoes [126]. Derilus and co-workers identified insecticide-specific gene expression patterns in *Ae. aegypti* from Puerto Rico. Using Illumina RNA-Seq technology, they could study these patterns associated with exposure to three insecticides, malathion, alphacypermethrin, and lambda-cyhalothrin. The mosquitoes were collected from two sites, Manatí and Isabela. They observed overexpression of cytochrome P450s across all resistant phenotypes. Some of the genes that were overexpressed were *CYP6Z7*, *CYP28A5*, *CYP9J2*, *CYP6Z6*, *CYP6BB2*, *CYP6M9*, and two *CYP9F2* orthologs [127].

Ingham and co-workers exposed a highly resistant *An. coluzzii* population from Burkina Faso to deltamethrin. They identified clear patterns in the transcriptomics data, wherein genes related to DNA repair, respiration, translation, behavior, and oxidoreductase processes were observed. From the study, they could conclude that the ageing and diel cycle has a profound impact on insecticide resistance [128]. Transcriptomics (RNAseq) coupled with WGS techniques revealed several changes at the transcriptome and the genome level of the major malaria vector *An. coluzzii* under resistance and susceptible states. Oxidative phosphorylation pathway genes were upregulated in the resistant population compared to that of the susceptible control population. Phenotypically, this observation translated to an increased and elevated metabolism of insecticides and thus resulted in the development of resistance [4].

Li and co-workers studied the role of GPCRs and related genes in insecticide resistance of the filarial vector *Cx. quinquefasciatus*. Several genes like *CPIJ014413*, *CPIJ019111*, *CPIJ007717*, and *CPIJ14419*, belonging to the class of GPCRs, were upregulated in response to permethrin. This study also revealed the existence of several downregulated GPCRs and related genes (*CPIJ003158*, *CPIJ003873* (beta-adrenergic receptor), *CPIJ003683* (5-hydroxy-tryptamine receptor 2B), *CPIJ003420*, *CPIJ007676*, *CPIJ017421*, and *CPIJ000647*) following permethrin exposure. They hypothesized that the downregulation of GPCRs and related genes in response to permethrin selection might have occurred as a homeostatic mechanism to balance the upregulation of GPCRs and related genes [129].

Yang and co-workers studied the differential expression pattern of P450s in *Cx. quinquefasciatus* when exposed to permethrin. They observed that several P450s’ gene expression was suppressed [125]. The downregulation of these P450s may have resulted from cellular responses to various endogenous and exogeneous compounds, directed to protect the cell from the negative effects of P450s-derived metabolism such as the generation of reactive oxygen species during the oxidation of nitric acid or arachidonic acid [130,131]. Additionally, downregulation might help to conserve energy molecules for complex transcriptional regulations and synthesis of other essential components during the response [130,132]. However, the exact reason for the downregulation of CYPs is poorly understood and needs further investigation. 

The meta-signature analysis studies compare and evaluate the intersection of hundreds of signature genes relevant to the underlying insecticide resistance mechanisms obtained through transcriptomic data. These intersected genes are the meta-signature genes for the underlying molecular mechanism of insecticide resistance. The meta-signature genes acquired across various pyrethroid-resistant mosquito populations in Africa yielded and identified several new insecticide-resistance genes [133]. The hexamerins, α-crystallins, and ATPase subunit-e were the three new gene families that may be involved in the binding and storage roles which hint at their possible potential role in the development of a sequestration-based insecticide-resistance mechanism [134,135]. When some of the members of α-crystallins were silenced using dsRNA in pyrethroid-resistant strains that were then exposed to control papers, there was no difference in mortality. However, when attenuation of the expression of a α-crystallin *AGAP007159* gene by RNAi was performed, significant mortality was observed following deltamethrin exposure, suggesting that α-crystallins are ubiquitously overexpressed and that *AGAP007159* was involved in altering the resistance phenotype of the strain. Whereas, for hexamerins, dsRNA-mediated attenuation of *AGAP001659* (one of the upregulated transcripts belonging to the class of hexamerins) resulted in a small but significant increase in mortality (43.5%). The meta-analysis identified an additional novel family of proteins, ATPase subunit-e. This transcript was the most highly overexpressed among all the *An. coluzzii* arrays studied. Suppressing the expression of the ATPase resulted in a significant increase in mortality post-deltamethrin exposure [133]. ATPases were previously thought to have a role in mitochondria, but they were also found to be in the midgut and salivary glands of insects, playing a role in lipid transport [136,137]. Most likely, they participate in the detoxification mechanism by generating energy molecules, adenosine triphosphate (ATP), for energy-intensive detoxification processes carried out by GSTs, esterases, and cytochrome P450s [138].

Transcriptional regulatory factors play a major role by modulating the expression of several genes involved in morphological diversification, developmental mechanisms, and insecticide resistance [139,140]. Sub-lethal exposures to insecticides have revealed the induction of large-scale transcriptomics changes, highlighting the complexity of insect–insecticide interactions. This necessitates further investigations to gain a better understanding [141]. Few studies have investigated the induction of multiple genes in response to insecticides, but the regulatory mechanisms underpinning these responses are still poorly understood. The majority of the significant vector mosquitoes lack known cis- or trans-acting regulatory components, and the function of the non-coding regulatory apparatus has received little attention in the literature. However, some studies have identified transcription factors and their pathways. The Nrf2-CNC pathway in vectors [142,143] and agricultural pests [144,145] as well as the ARNT-AhR pathway in agricultural pests [140,146] have been studied. However, the need for a comprehensive transcriptomic regulation factor analysis still remains. *Maf-S*, a transcription factor located in the nucleus, controls several genes that are involved in xenobiotic defense. The transcriptomics dataset has revealed that expression of the *Maf-S* factor has been shown to correlate with the expression of several insecticide-resistance candidate genes in *An. gambiae*, like the key insecticide metabolizers *CYP6M2* and *GSTD1.* When attenuation of this transcription factor was carried out through RNAi, there was significant downregulation of the effector molecules, and mortality increased significantly with insecticide exposure [143]. While studying the resistant transcriptomics database of *Ae. aegypti*, Bottino-Rojas and co-workers found that approximately 70% of the upregulated transcripts in these populations represented ARE1 and/or ARE2 (ARE-Antioxidant Response Element) in their promoters. The implication of this study is that these cis-regulatory elements may serve as relevant genomic markers for the resistance phenotype in natural populations [142].

Sensory appendage protein 2 (SAP2) is a type of chemosensory protein found in the legs of African *An. gambiae*. It was found to have a significant role in the development of insecticide resistance against synthetic pyrethroids. It was observed that *SAP2* expression increased with exposure to pyrethroids. Silencing of the *SAP2* gene through RNAi fully restored the mortality of the mosquito. It was found to have a high binding affinity towards the pyrethroid class of insecticides and had a sequestration-based mechanism towards the development of insecticide resistance [147].

At the whole-genome level, Ying Li and co-workers systematically identified the trypsin superfamily of genes and examined their diversity, traits, and evolutionary connections in *An. sinensis* and other insects. *An. sinensis* was found to harbor three hundred and forty-two trypsin genes, which were categorized into seven groups according to phylogenetics, homology, and the presence of distinctive domains. These genes were upregulated, and they were identified based on their insecticide-resistance characteristics in the pyrethroid-resistant populations. RNA interference (RNAi)-based knocking down of these genes decreased the pyrethroid resistance of *An. sinensis*. Four specific trypsin genes (*ASTRY2B*, *ASTRY43A*, *ASTRY90*, and *ASTRY113C*) were found to be associated with pyrethroid resistance based on transcriptome analyses of three field-resistant populations, with qRT-PCR validation. Serine proteases like trypsins play roles in various biological processes like digestion and the immune response. This class of serine proteases is upregulated in resistant conditions, suggesting that they play a role in the degradation of pyrethroids or in the mitigation of their toxic effects. Understanding these genes will help in comprehending molecular mechanisms underlying resistance development. The management of resistance development and vector control will be possible by designing specific inhibitors targeting these specific trypsins. Future research is essential to explore their bio-molecular interactions with pyrethroids to further elucidate their roles in resistance [148].

The following table (Table 3) explores some of the transcriptomics works and lists the differentially expressed genes as seen in transcriptomics data.

Some of the studies depicted in Table 3 shed light on various underlying transcriptomic profiles in insecticide resistance. The cytochrome P450 family of enzymes plays a very crucial role in the detoxification of several insecticides. Some of the major *CYP* genes that were observed in detoxification belonged to the classes *CYP6*, *CYP9*, *CYP4* and *CYP12*. The wider diversity of the *CYP* family of genes indicates a broader metabolic capacity of these genes for the detoxification of insecticides. Multiple genes belonging to the family of *CYP6*, like CYP6Z7, *CYP6BB2*, *CYP6M2*, *CYP6P3*, and *CYP6Z18*, are observed to have important functions in the degradation of pyrethroids, organophosphates, and carbamates [127,149,150]. Some of the genes belonging to the family of *CYP9* (*CYP9J28*, *CYP9J32*, and *CYP9K1*) were also observed and are important players in pyrethroid resistance [81,153]. Multiple genes belonging to the family of *CYP4* and *CYP12* [122,123,133] are also observed across species in transcriptomics data. GSTs (*GSTD1*, *GSTMS3*, etc.) and carboxylesterases are found to act as secondary detoxification players across all species. Unique downregulation and upregulation patterns are observed in all the studies. These patterns may be time-dependent for the phased detoxification response, metabolic adjustments, and enhancement of physical barriers for blocking the entry of insecticides. The difference in the level and pattern of expression of the respective genes can also vary by various factors like life stage (larva/adult), age, exposure history, species, and region of the body which is exposed [125,129,149,154]. Beyond metabolic enzymes, genes related to the structural response (cuticular proteins), stress response (HSPs: Heat shock proteins), and signal transduction (GPCRs: G-protein coupled receptor) broadly indicate that resistance mechanisms can comprise both metabolic and non-metabolic adaptations [129,155].

While most studies concentrate on upregulated transcripts, it is important to understand the importance of downregulated transcripts while analyzing the transcriptomics data. The downregulated transcriptomics data are often overlooked despite their importance in understanding processes and responses. Upregulation is often considered to be the standard for measuring the response to stimuli like stress, infection, and insecticide exposure since the expression is visibly magnified in upregulated conditions. They are often seen as active participants in biological processes and have more visible roles in defense mechanisms and metabolic processes. This bias towards positive associations often results in overlooking downregulated genes. The downregulated transcriptomics data may essentially contain the details about many genes like CYPs, GPCRs, HSPs, etc., which are involved in metabolic and non-metabolic activities. The underexpression of certain genes might be part of a genetic adaptation, where upregulation of some resistant pathways may lead to downregulation of others to balance energy expenditures [156]. Upregulation of HSPs may be involved in the cellular stress response under insecticide exposure. These HSPs may function in stabilizing, folding, and degrading damaged proteins resulting from oxidative stress during insecticide exposure [157]. While the downregulation of HSPs is not widely studied, they may be involved in optimizing energy allocation to complex processes, apoptosis modulation, and fine-tuning of stress signals. They can also modulate the upregulation and downregulation of P450s and other resistance-associated genes which are evident across resistant and susceptible vector populations [155].

However, it is very important to conclude that underexpression transcriptomics data are as vital as overexpression data for a complete understanding of resistant phenotypes and unravelling complex resistance mechanisms in vector species.

### 2.4. Proteomics

Proteins as effector molecules are vital for executing many biological processes affecting the biological and physiological response. Proteomics is a versatile tool that has been used to study the development of resistance in insects [158]. The recent advances in the field of proteomics have assisted entomologists in looking beyond genomics. Proteomics has paved the way for improving the understanding of mosquito–insecticide interactions and the subsequent development of resistance in mosquitoes to insecticides. It may be possible that genes that are not associated with insecticide resistance may be observed in transcriptomics studies. It is, therefore, necessary to be cautious in the interpretation of differentially expressed genes. They may have a direct or indirect effect or may not be involved with resistance development. This could be due to various factors, such as (i) genetic drift, which occurs due to a gene‘s location relative to another gene that may be undergoing positive selection, (ii) a common regulatory process, which may correlate with up- or downregulation of resistance genes, and (iii) differential changes in response to biotic or abiotic factor changes that occur when resistance genes are expressed. Hence, transcription may not necessarily capture the actual event when mosquitoes are stressed due to insecticides. Therefore, it is important to understand and investigate expression dynamics at the protein level [159]. Therefore, proteomics analysis is important to complement and validate transcriptomics data.

Global proteomic profiling for cypermethrin, propoxur, and dimethyl-dichloro vinyl-phosphate-resistant *Cx. pipiens pallens* against the susceptible *Cx. pipiens pallens* strain indicated that multiple resistant mechanisms were found to operate simultaneously in resistant strains of mosquitoes. Mechanisms such as lower penetration, the sequestration of insecticides, increased biodegradation of the insecticides through subtle alterations in cuticular protein levels, and enhanced activities of detoxification enzymes belonging to the class of P450s and glutathione S-transferases have been observed [56]. Elevated resistance levels to cypermethrin were also observed in insecticide-selected strains of *Cx. pipiens pallens*. Proteomics analysis through parallel reaction monitoring detected almost 2892 proteins out of which 2885 proteins were differentially expressed and showed quantitative significance in four stages (egg, larvae, pupae, and adult) of *Cx. pipiens pallens* cypermethrin-resistant and -susceptible strains. Significant enrichment of proteins was found in several pathways and local expressions which included cuticular proteins, insecticide detoxification pathways (cytochrome P450, glutathione S-transferase, esterase, and ATP-binding cassette), as well as pathways like oxidative phosphorylation and hippo signaling. These large sets of proteins were involved in various cellular and metabolic pathways, and they were directly and indirectly involved with insecticide resistance by possibly influencing cell physiological activities like the detoxification of insecticides and transcriptional regulation [160].

The molecular basis of cytochrome P450 monooxygenases-mediated pyrethroid resistance was studied in CKR (CYP + KDR: ROCK) and SP (Singapore strain) strains of *Ae. aegypti*. A combination of transcriptomics and proteomics was applied to decipher the CYPs mode of resistance in the resistant strains. The studies revealed that most of the CYPs that were overexpressed in the CKR-resistant strain were due to trans-acting factors via upregulating factors increasing the expression of one or more CYPs. Around 65 transcription factors or long non-coding RNAs (lncRNAs) were identified. The molecular basis of CYP-mediated resistance in the SP strain was narrowed down to three plausible hypotheses based on (1) non-synonymous mutation (transcription factors or lncRNA) leading to overexpression of CYPs, (2) mutations in the promoter of a switch, or (3) stabilization of CYP proteins [156].

iTRAQ-based quantitative proteome analysis was used to identify the proteins linked to pyrethroid resistance in *Cx. pipiens pallens*. Numerous proteins were found that were not previously believed to be connected to pyrethroid resistance. These results imply that the nature of molecular mechanisms in insecticide resistance development in mosquitoes is complex. There is an important need for identifying and classifying these mechanisms to better understand resistant vector control. A protein classified as CYP6AA9 belonging to the family of CYP6 was found to be overexpressed in the lab deltamethrin-resistant strains. Further, the differentially expressed protein was found to express at different stages (fourth instar, larvae, pupae, and adult) of lab-resistant strains when compared to that of susceptible strains [161].

Quantitative proteomics analysis of permethrin-resistant adult mosquitoes and temephos-resistant larvae of *Ae. aegypti* revealed the presence of 501 and 557 differentially expressed proteins, respectively. Proteins such as NADPH cytochrome p450 reductase, CYP9J9, CYP9J27, cytB, motor-related proteins like myosin, and several isoforms of glutathione–S-transferases isoforms were identified among the differentially expressed proteins. Some of the studies revealed that the cytochrome c oxidase class of enzymes was upregulated. These enzymes are involved in the production of ATP via oxidative phosphorylation. As discussed earlier, ATP is the energy currency that is required for many cellular functions involved in the respiration and development of insecticide resistance (activation of detoxification systems). A 1.5-fold increase in the expression of *cytB* was observed in the strains resistant to permethrin and temephos [162].

Proteomics is emerging as an important tool to study insecticidal resistance in mosquitoes. It is crucial for understanding and mapping resistance pathways. It can be used to identify key biomarkers and to study immunity, energy allocation, and stress responses in resistant mosquitoes.

### 2.5. Metabolomics

Metabolomics involves the methodological study of complex cellular processes of a biological system by analyzing its entire metabolome (all measurable metabolites) under a given set of conditions [163]. Mosquitoes involve complex metabolic pathways that take part in the detoxification of insecticides. Many of these specific metabolic pathways are still unclear [161]. Li and co-workers classified metabolic pathways and differential metabolites that were enriched in deltamethrin-exposed susceptible and resistant *An. sinensis* (larval and adult stage) mosquitoes. A pathway enrichment analysis in the KEGG (Kyoto Encyclopedia of Genes and Genome) database revealed the enrichment of amino acid metabolism pathways (tryptophan, glycine, serine, and threonine) and amino acid biosynthesis pathways (phenylalanine, tyrosine, and tryptophan) in resistant *An. sinensis* larvae, whereas some of the pathways were specifically enriched in resistant *An. sinensis* adults. These pathways included ABC transporters, starch and sucrose metabolism, pentose and glucuronate interconversions, glycerol-phospholipid metabolism, and ether lipid metabolism. These pathways were related to energy metabolism and energy changes happening after exposure to insecticides. Most of the differential metabolites that were observed between the susceptible and resistant mosquitoes belonged to the class of organo-oxygen compounds, glycerol-phospholipids, and purine nucleotides synthesis which were crucially important for protein synthesis, DNA replication, and energy production. However, resistant strains exhibited a predominance of amino acid metabolism and biosynthesis pathways, which was supported by multiple studies involving pesticide exposure and an increase in amino acid concentration following the exposure [164,165]. Glycerophospholipid metabolism was proposed as a potential target for understanding deltamethrin resistance and management owing to its potential role in membrane integrity and lipid biosynthesis [166].

An untargeted NMR-metabolomics approach was used to study the metabolic profiling of *Ae. aegypti* responses to temperature and insecticides like DDT (4%), malathion (5%), and deltamethrin (0.05%). When exposed to insecticides under the control of specific temperatures, metabolites like pyruvate, maltose, citrate, nicotinate, and β-hydroxybutyrate were identified [167]. These metabolites were part of energy-generating pathways, i.e., glycolysis/gluconeogenesis, and the tricarboxylic acid cycle [168]. They are essential during biotic/abiotic stress [169] and flying [170]. Choline and lipids were also identified, and they are associated with insecticide susceptibility/resistance [171] and thermal tolerance [172]. In the oxidative phosphorylation pathway, genes were upregulated in the resistant population compared to that of the susceptible control population. Phenotypically, this observation translated to increased and elevated metabolism of insecticides and thus resulted in the development of resistance [4].

The application of metabolomics in studying insecticide resistance in mosquitoes is important, yet very few studies have been conducted to date. Metabolomics provides valuable information on pathways regulated by mosquitoes to support their survival under insecticidal stress. It also gives important information on the fitness cost of mosquitoes associated with resistance and aids in understanding how mosquitoes allocate energy for reproduction, survival, and growth.

### 2.6. Epigenomics

Changes in the regulation of gene activities that occur independently of or without alterations in gene sequences are referred to as epigenomics. This area of omics studies a complete set of all epigenetic modifications on the genetic material of the cell. The epigenomics field is analogous to the other omics fields like genomics and proteomics. Epigenetics involves DNA and histone modifications and thus plays a role in the regulation of gene expression, and the process happens without altering the DNA sequence [173]. The reprogramming of gene expression changes the response of mosquitoes to environmental stimulatory factors like climate change and insecticide exposure. The contribution of epigenetic mechanisms in the rapid evolution and development of insecticide resistance in mosquitoes is poorly understood [59]. Very few studies have been conducted in this area. Oppold and colleagues reported that changes in global DNA methylation levels in the Asian tiger mosquito, *Ae. Albopictus*, were correlated with decreased insecticide sensitivity to the neonicotinoid imidacloprid at the phenotypic level following exposure to various stimuli [174].

### 2.7. Microbiomics

Microbiomics or microbiota profiling refers to the study of microorganisms within a given community, investigating their potential roles in various biological functions. In addition to its crucial role in viral infectivity and protection against parasites [175], the gut microbiota of mosquitoes has shown to mediate the development of insecticide resistance [50]. Insecticide-degrading gut bacteria have been observed in both agricultural pests and mosquitoes [176,177]. Most of the microbiota are acquired from aquatic sources during the larval stage of mosquitoes and also depend on the food source of adults [178,179].

The connection between mosquito symbiotic bacteria and insecticide resistance has garnered more and more attention owing to the capability of symbionts to degrade insecticides. Mosquitoes belonging to the species of *An. stephensi*, *An. Arabiensis*, and *An. albimanus* have shown a connection between the prevalent microbiota and their resistance characteristics towards the pyrethroid class of insecticides [50,180,181,182]. Dada and colleagues explored the relationship between the microbiota of the *An. albimanus* species and its resistance to the organophosphate insecticide fenitrothion. They observed enrichment of *Klebsiella pneumoniae* and other organophosphate-degrading bacteria in resistant strains. Additionally, a differential abundance of bacterial xenobiotic-degrading enzymes, particularly carboxylesterases and phospho-monoesterases, was noted in resistant strains compared to susceptible groups [50]. *Myxococcus*, a bacterial species known to produce chitinases [183], bioactive antifungal agents, and certain inhibitors of cellular respiration [184], was abundantly found in the susceptible class, whereas differential abundance of microbiota belonging to species like *Sphingobacterium*, *Lysinibacillus*, *Streptococcus*, and *Rubrobacter* was found in the resistant mosquito population of *An. gambiae* [185]. The microbiota of *An. coluzzii* was studied by Pelloquin and co-workers in response to deltamethrin exposure. They could prove that the resistant and susceptible groups differed significantly from one another in microbiota diversity. Insecticide-degrading taxa (*Ochrobactrum*, *Lysinibacillus*, and *Stenotrophomonas*) were more abundant in the resistant population (2–3 days old group) compared to the susceptible group (5–6 days old), which showed an overabundance of *Serratia* and *Asaia* taxa [186]. The higher abundance of insecticide-degrading bacteria in the resistant population suggests that these microbes may confer an adaptive advantage by breaking down insecticides, thereby reducing their toxicity. Previous investigations have shown that mosquito microbial diversity declines with age, which correlates with a decrease in insecticide resistance [187,188,189] in older mosquitoes.

Soltani and co-workers found a direct correlation between temephos resistance and the presence of bacteria in *An. stephensi* symbiotic organs. When antibiotics were introduced, the resistance profile of the strain shifted to a susceptible status, demonstrating that eliminating bacterial symbionts in a resistant population could artificially break resistance to temephos in *An. stephensi* [182].

The relationship between the obligatory intracellular endosymbiont *Wolbachia* and the resistance of vectors to insecticides has been explored. In a study by Shemshadian and co-workers, a negative correlation between *Wolbachia* bacterial density and resistance was observed in *Cx. quinquefasciatus*. The *Wolbachia*-infected population showed reduced resistance against deltamethrin. However, it remains unclear whether the resistance reductions in the vectors are entirely due to the high density of these endosymbionts or are also influenced by resistance genes. Some studies suggest that *Wolbachia* endosymbionts are partly responsible for the fitness cost of insecticide resistance; however, the resistance genes particular to different strains can still play roles in the development of insecticide resistance, and thus, an association between fitness cost and resistance genes exist even in the *Wolbachia*-free population. *Wolbachia* infection, therefore, induces synergistic additional resistance costs for some specific traits [190].

The symbionts also play an important role in affecting the vectorial capacity and altering the physiology of the host mosquitoes [191]. For example, *Asaia* spp. was shown to activate the expression of antimicrobial peptides in *An. stephensi* [192], while a few strains of *S. marcescens* inhibited *Plasmodium* parasite development by altering the immunity-related effector genes *TEP-1* and FBN9 [193]. SmEnhancin, an enzyme produced by *S. marcescens*, digests the gut epithelia mucins and facilitates virus penetration in the gut. This event makes the mosquito susceptible to virus infection [6]. A similar mechanism may occur in mosquitoes by the action of some proteins that the *Serratia* species express. These proteins may increase the permeability of mosquito internal organs to deltamethrin, eventually making the mosquito susceptible to insecticides [186].

These examples highlight the increasing importance of studying microbiomics, as it provides a comprehensive approach to understanding the multifactorial and synergistic nature of resistance mechanisms in vector mosquitoes.

## 3. Discussion

We have outlined several molecular studies that utilize omics approaches to investigate insecticide resistance. However, further research is needed to deepen our understanding of resistance mechanisms, particularly in the less-explored fields of epigenomics, proteomics, and metabolomics. The existing literature in these areas remains limited. Integrating research on insect vectors with advanced proteomics and genomics tools is critical to unravelling the emergence of insecticide resistance. Genomic studies have identified several resistance-related mutations and genes, while transcriptomic research has illuminated the dynamic changes in gene expression influenced by insecticide exposure and environmental factors.

Functional characterization of these resistance genes in mosquitoes using advanced techniques such as CRISPR and RNA interference (RNAi) can validate their roles and mechanisms with greater precision. Such insights are essential for developing sustainable vector control and surveillance strategies. Moreover, the role of the mosquito gut microbiome in insecticide resistance is increasingly recognized. Altering the gut microbiota of mosquito populations to reduce insecticide-degrading bacteria holds significant potential. Research is also needed to understand the metabolites produced by the microbiome and their influence on mosquito fitness. Investigating microbial community shifts across the mosquito life cycle is crucial for correlating these changes with resistance levels.

The integration of omics data for building resistance prediction models offers a powerful approach to identifying molecular markers associated with resistance in vector mosquitoes. However, achieving high accuracy in data annotation is essential to ensure the reliability of these predictive models. Available omic tools like scCross, PathIntegrate, and CustOmics are a few of the tools which have been recently designed to integrate omics data and study transcriptomics data. This information can help in the construction of gene regulatory networks, whereas proteomics can be useful in mapping protein–protein interactions and identifying key nodes in insecticide-resistance cellular networks [194,195,196,197,198].

While omics approaches are invaluable for understanding insecticide resistance, they come with certain limitations. The relationship between phenotypic traits and omics data often requires rigorous evaluation in the laboratory. Although omics can identify novel resistance markers, functional validation remains crucial to confirm their roles. Insecticide resistance in vector mosquitoes is a complex, multifactorial process involving synergistic interactions. The link between genetic data and observable traits is especially important. For instance, single-nucleotide polymorphisms (SNPs) identified in genomic data must be validated to establish their association with resistance phenotypes. SNPs present in heterozygous conditions can reverse in the absence of insecticide exposure, leading to reduced resistance levels [199]. This reversal is often attributed to the high fitness cost of heterozygous-resistant alleles, which are less stable compared to homozygous-resistant or susceptible genotypes [79,200]. Transcriptomics, while generating substantial data on differential gene expression, does not always align with the protein abundance profiles observed in proteomics. Discrepancies arise due to factors such as post-translational modifications, protein turnover rates, technical variability, and the complexities of data integration. For example, Sun et al. reported inconsistencies between RNA-seq and proteomics data. Overexpression of several cytochrome P450 genes was observed in both transcriptomic and proteomic datasets. Five CYPs were found to be overexpressed only at the protein level, underscoring the role of post-translational modifications and protein stabilization in resistance mechanisms [156]. Another challenge is the cost of carrying out omics experiments. It should be affordable for large-scale applications. Despite these hurdles, the insights gained from omics studies are invaluable. To maximize their utility, it is essential to strike a balance between resource allocation and addressing these limitations effectively.

## 4. Conclusions

Globally, vector control efforts rely heavily on the use of synthetic insecticides. However, the continuous exposure of mosquito populations to various physical and biological factors drives evolutionary adaptations, ultimately leading to the development of insecticide resistance. This growing resistance poses significant challenges to the effective control of mosquito populations and, by extension, the management of vector-borne diseases such as malaria, dengue, and zika.

To address this issue, the application of omics technologies—such as genomics, transcriptomics, proteomics, and metabolomics—has proven invaluable in studying the mechanisms underlying insecticide resistance in mosquitoes. These approaches provide detailed insights into the genetic, molecular, and biochemical pathways that contribute to resistance, enhancing our ability to devise more effective vector control strategies. However, a comprehensive understanding of insecticide resistance requires integrating omics data with systems biology. Systems biology allows for the exploration of interactions across multiple biological scales, providing a holistic view of how resistance mechanisms develop and persist in mosquito populations. This integrative approach can enrich our knowledge of how various factors, including environmental pressures and mosquito biology, interact to drive resistance.

Developing quantitative predictive models is another critical component in controlling insecticide resistance. These models can incorporate key variables such as insecticide dosage, timing, and frequency of application, genetic mutagenicity, fitness costs, and reproductive success (fecundity). Such models are instrumental in anticipating the emergence and spread of resistance mechanisms. Additionally, they can help assess the potential impact of resistance on the mosquito’s competence to transmit pathogens, enabling more targeted and sustainable interventions.

By embedding these predictive models into mosquito surveillance and public health programs, we can significantly enhance our capacity to monitor and mitigate the spread of resistance. This integration can also facilitate research aimed at identifying novel resistance markers and designing alternative vector control strategies. Ultimately, this comprehensive approach can transform vector control efforts, improving outcomes for public health and reducing the burden of vector-borne diseases on global communities.

## Figures and Tables

**Table 1:** The non-exhaustive list of major vector-borne diseases is arranged in the following table below with the respective vector and type of pathogen responsible [22].

Vector	Disease	Pathogen
*Aedes*	Chikungunya	Chikungunya Virus
	Dengue	Dengue Virus
	Lymphatic filariasis	*Wuchereria bancrofti*, *Brugia malayi*
	Rift valley fever	Rift Valley Fever Virus
	Yellow fever	Yellow Fever Virus
	Zika	Zika Virus
*Anopheles*	Malaria	*Plasmodium falciparum*
	Lymphatic filariasis	*Wuchereria bancrofti*, *Brugia malayi*
*Culex*	West Nile fever	West Nile Virus
	Japanese encephalitis	Japanese encephalitis Virus
	Lymphatic filariasis	*Wuchereria bancrofti*, *Brugia malayi*

**Table 3:** Differential gene profiling/transcriptomic data obtained in different mosquito vectors under insecticide-exposed conditions.

Scientific Name	Genes Detected as Overexpressed in Transcriptomics Study with Exposure to Insecticides	Ref.
*Aedes aegypti*	(a) Upregulated: *CYP9J10*, *CYP9J9*, *CYP9J27*, *CYP9J26*, *CYP9J28*, *CYP6BB2*, ABC transporter genes, and odorant-binding proteins genes Downregulated: *CYP6AH1*, *CYP4J16*, aldo keto reductase, carboxylesterase, and NADPH cytochrome P450	[39,149]
	(b) Upregulated: *CYP9M4*, *CYP9M5*, *CYP9M6*, *CYP6Z7*, *CYP6Z8*, *CYP6BB2*, *CYP6F2*, *CYP6F3*, and *cytochrome B5*	[39,81]
	(c) Upregulated: *CYP9J9*, *CYP9J28*, *CYP9J32*, 8 other P450 genes, 2 GST genes, 2 CCE genes, and aldo-keto reductase	[39]
	(d) Upregulated: *CYP6M11*, *CYP6N12*, *CYP6F3*, and 1 UGT Gene	[39,150]
	(e) Upregulated: *CYP6Z7*, *CYP28A5*, *CYP9J2*, *CYP6Z6*, *CYP6BB2*, *CYP6M9*, *CYP9F2*; *CYP6BY1* and carboxylesterase gene: unique to malathion resistance Downregulated: *CYP325N2*, *CYP6N15*, and *GSTS1*; *GSTD1*: unique to alpha-cypermethrin resistance; *CYP4H29*, *CYP307A1*, *CYP6P12*, and *GSTE6*: unique to lambda-cyhalothrin resistance	[127]
	(f) Upregulated: *CYP6M11*, *CYP4D38*, *ESTERASE B1*, *CYP4C38*, *GPHX1*, microsomal GST and ABC transporter genes, several Heat Shock Proteins (HSPs), *CYP9J6*, *CYP9J9*, and *CYP9J31* Downregulated: *CYP9J22* (6th hour), *GSTT4* (10th hour), ATP-binding genes, GTPase activity, protein processing, signal transduction, and spermidine biosynthesis (24th h)	[122]
*Anopheles gambiae*	(a) Upregulated: cytochrome P450 genes (*CYP6Z3*, *CYP4C28*, *CYP12F2*, and *cytochrome B5*); GSTS *(GSTD1*, *GSTD7*, *GSTD3*, *GSTE5*, and *GSTMS3*); carboxylesterase genes (*COEAE8O*); hexamerins and ATPase subunits Downregulated: transcripts enriched in vitellogenin and lipoprotein domains	[133]
	(b) Upregulated: *SAP2*	[147]
	(c) Upregulated: cytochrome P450s (*CYP12F2*, *CYP12F3*, *CYP4H15*, *CYP4H17*, *CYP6Z3*, *CYP9K1*, *CYP4G16*, and *CYP4D17*); cuticular proteins (*CPR30*, *CPR130*, *CPR15*, *CPR16*, *CPR76*, *CPAP3-A1B*, and *CPAP3-A1C*); salivary gland proteins (*SG9*, *AGAP011460*, and *AGAP009473*); carboxylesterase genes (*COEJHE5E* and *COE22933*) and glutathione S-transferases (*GSTE2* and *GSTMS3*)	[123]
	(d) Upregulated: cytochrome P450 (*CYP6Z2* and *CYP6M2*); GSTs (*GSTS1-2*); superoxide dismutase (*SOD3B*)	[151]
	(e) Upregulated: cytochrome P450 genes (*CYP6P3* and *CYP6Z2*); rhodopsin receptor gene, acetylcholinesterase, and GABA receptor Downregulated: *GSTs* (*GST1-5*, *GSTE3*, *GSTE4*, *GST1-6*, and *GSTU1*); cytochrome oxidase (*CYP6AK1*)	[126]
	(f) Upregulated: multiple cytochrome P450 genes	[152]
*Culex quinquefasciatus*	(a) Upregulated: cytochrome P450 gene family *CPIJ020018* (*CYP6Z16* and *CYP6Z18*) and *CYP6N23*; GSTs and esterases	[124]
	(b) Upregulated: GPCRs (*CPIJ014413*, *CPIJ019111*, *CPIJ007717*, and *CPIJ14419*) Downregulated: GPCRs (*CPIJ003158*, *CPIJ003873* (beta adrenergic receptor), *CPIJ003683* (5-hydroxy-tryptamine receptor 2B), *CPIJ003420*, *CPIJ007676*, *CPIJ017421*, and *CPIJ000647*)	[129]
